# Laparoscopic Resection of a Mesorectal Solitary Fibrous Tumor: A Case Report and Review of the Literature

**DOI:** 10.7759/cureus.113009

**Published:** 2026-07-20

**Authors:** Atsushi Sugimoto, Hiroshi Tsuchihashi, Hiroyuki Fujimoto, Masayasu Kawasaki

**Affiliations:** 1 Department of Surgery, Bellland General Hospital, Osaka, JPN

**Keywords:** laparoscopic surgery (lp), mesenchymal tumors, minimally invasive surgery, pelvic soft tissue tumor of unknown origin, solitary fibrous tumor (sft)

## Abstract

Solitary fibrous tumor (SFT) is a rare mesenchymal neoplasm that can arise in various anatomical sites, whereas occurrence in the mesorectum is extremely uncommon. We report a case of mesorectal SFT successfully treated by laparoscopic resection and review the literature. A 62-year-old male was referred for further examination of a pelvic mass incidentally detected on computed tomography (CT). Imaging revealed a well-circumscribed 35-mm tumor located between the lower rectum and prostate without local invasion or distant metastasis. Although mesenchymal tumors such as gastrointestinal stromal tumor (GIST), schwannoma, and SFT were considered, a preoperative diagnosis could not be established due to biopsy difficulty and potential risks. Laparoscopic resection was performed for both diagnostic and therapeutic purposes. Histopathology revealed spindle cells in a patternless pattern with prominent vascularity. Immunohistochemistry showed positivity for CD34 and Bcl-2, and the morphological and immunophenotypic findings were considered consistent with SFT, although STAT6 immunostaining was not available. The postoperative course was uneventful, with no recurrence after six months of follow-up. In selected cases, minimally invasive surgery (MIS) enables complete tumor resection with organ preservation, while long-term follow-up remains essential due to the risk of late recurrence.

## Introduction

A solitary fibrous tumor (SFT) is a rare mesenchymal fibroblastic neoplasm accounting for less than 2% of all soft tissue tumors [1]. This clinical entity was first described by Klemperer and Coleman in 1931 [2]. SFT was originally described as a pleural tumor but is now known to arise in various extrathoracic sites, including the head and neck, abdominal cavity, retroperitoneum, and extremities [3]. The NAB2-STAT6 fusion gene has been identified as a characteristic genetic alteration of SFT, and nuclear STAT6 immunohistochemistry has been reported as a highly sensitive and specific diagnostic marker. However, SFT arising in the mesorectum is extremely rare, and only a limited number of cases have been reported. Mesorectal SFT presents a diagnostic challenge because preoperative tissue diagnosis is often difficult, and the optimal surgical approach remains uncertain. In particular, the role of minimally invasive surgery (MIS) has not been well established. Although SFTs typically exhibit an indolent clinical course, they possess intermediate malignant potential with a risk of local recurrence and distant metastasis, underscoring the importance of accurate diagnosis, complete surgical resection, and long-term follow-up. Here, we report a rare case of mesorectal SFT successfully treated by laparoscopic tumor resection and discuss the clinical decision-making process, including diagnostic considerations and surgical indications, with a review of the literature.

## Case presentation

**Figure 1:**
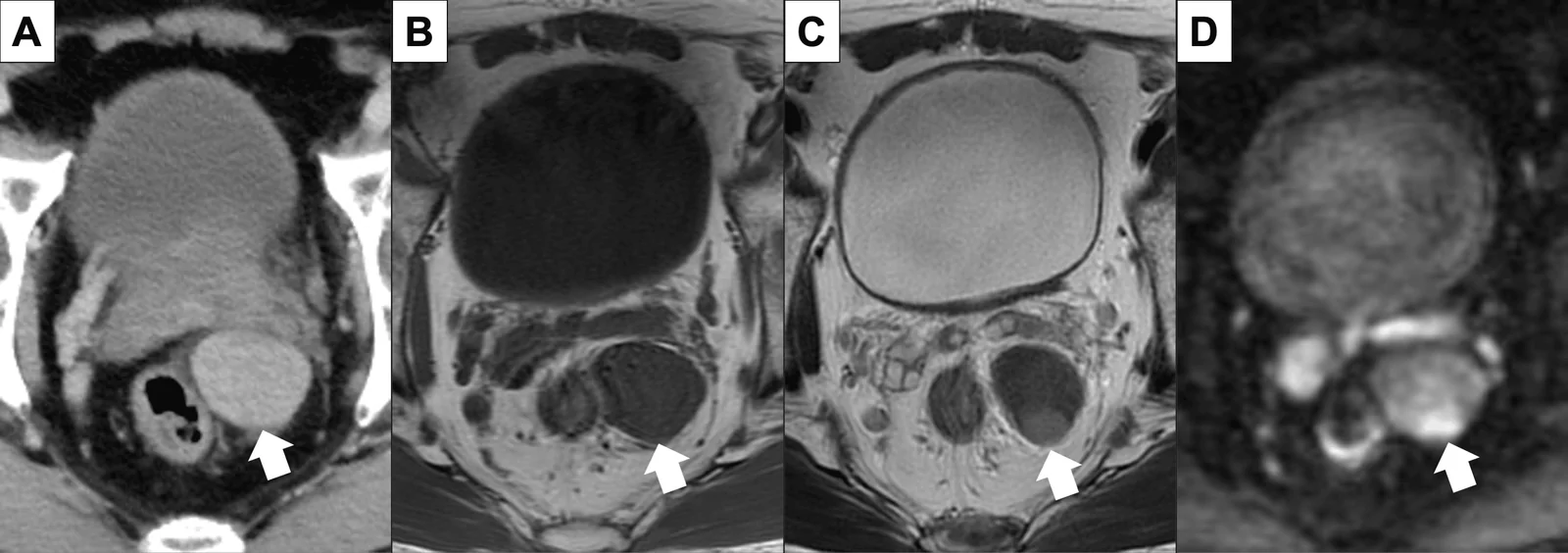
Abdominal computed tomography (CT) and magnetic resonance imaging (MRI) findings (A) Contrast-enhanced CT identifies a well-circumscribed, 35-mm tumor located between the lower rectum and the prostate. (B) T1-weighted MRI reveals a smooth-surfaced mass with low signal intensity. (C) T2-weighted MRI shows mixed low and isointense signals. (D) Diffusion-weighted imaging (DWI) exhibits isointense to focal high-signal intensity. Arrows indicate the tumor.

A 62-year-old male was referred to our hospital for the examination of a perirectal mass incidentally detected by computed tomography (CT) at a previous hospital. He was asymptomatic. His physical examination was unremarkable, with a height of 158 cm, a weight of 58 kg, and a body mass index (BMI) of 23.2 kg/m2. Digital rectal examination revealed a firm, elastic, palpable mass on the left anterior aspect of the rectum. Laboratory investigations were within normal limits. The levels of the tumor markers carcinoembryonic antigen (CEA), carbohydrate antigen 19-9 (CA19-9), and soluble interleukin-2 receptor (sIL-2R) were 2.9 ng/mL, 13 U/mL, and 342 U/mL, respectively, all of which were within their reference ranges. Colonoscopy confirmed no mucosal abnormalities in the rectum. Contrast-enhanced CT identified a well-circumscribed, 35-mm tumor located between the lower rectum and the prostate, with no evidence of significant lymphadenopathy or distant metastasis (Figure 1, Panel A). Magnetic resonance imaging (MRI) revealed a smooth-surfaced mass with low signal intensity on T1-weighted images (Figure 1, Panel B). T2-weighted images showed mixed low and isointense signals (Figure 1, Panel C), while diffusion-weighted imaging (DWI) exhibited isointense to focal high-signal intensity (Figure 1, Panel D).

**Figure 2:**
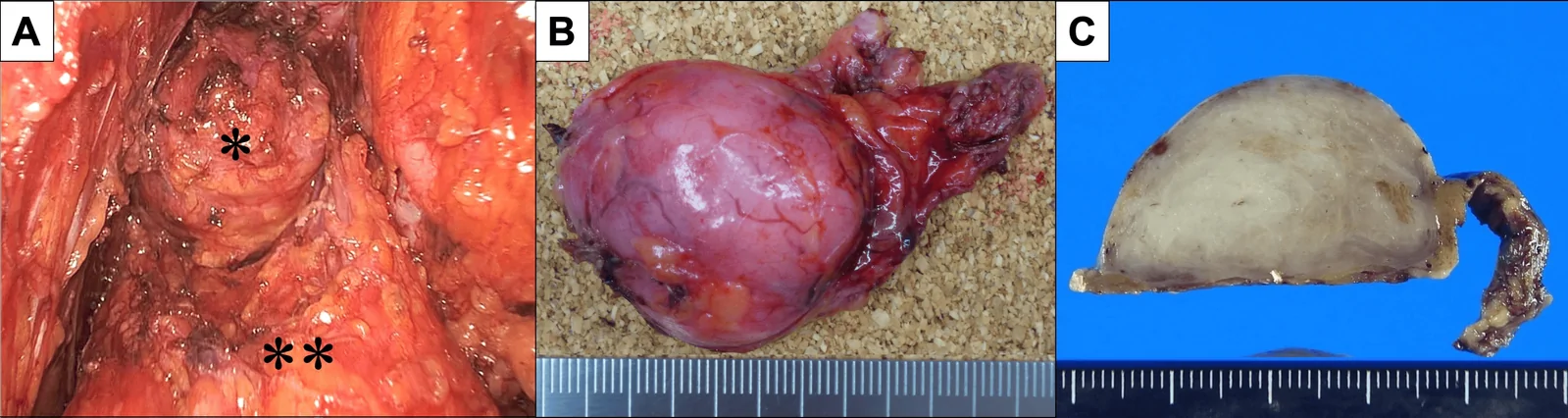
Operative and macroscopic appearances (A) The tumor is located in the mesorectum without invasion into the adjacent organs (* tumor, ** rectum). (B) Macroscopically, the resected specimen was a well-circumscribed solid tumor measuring 35 mm in diameter.

There was no definitive evidence of direct invasion into the rectum or prostate. Based on these findings, mesenchymal tumors such as gastrointestinal stromal tumor (GIST), schwannoma, leiomyoma, and SFT were considered. A well-circumscribed, hypervascular pelvic mass without evidence of adjacent organ invasion, together with characteristic MRI findings, should prompt consideration of SFT among the differential diagnoses. Preoperative biopsy was deemed technically difficult due to the tumor location and risk of complications. Therefore, surgical resection was planned for both diagnostic and therapeutic purposes. Laparoscopic tumor resection was performed. The tumor was located in the lower mesorectum, showing no invasion into the adjacent rectum or prostate (Figure 2, Panel A). Complete tumor resection with preservation of the rectum was achieved. The total operative time was 279 minutes with minimal blood loss (3 mL). Despite the relatively small size of the tumor, the operative time was prolonged because precise dissection was required within the restricted workspace of the deep pelvis to preserve the rectum, prostate, and surrounding neurovascular structures while maintaining an intact surgical margin. The patient's postoperative course was uneventful, and he was discharged on postoperative day 11. Macroscopically, the resected specimen was a well-circumscribed solid tumor measuring 35 mm in diameter (Figure 2, Panel B). Complete (R0) resection was confirmed with negative surgical margins.

**Figure 3:**
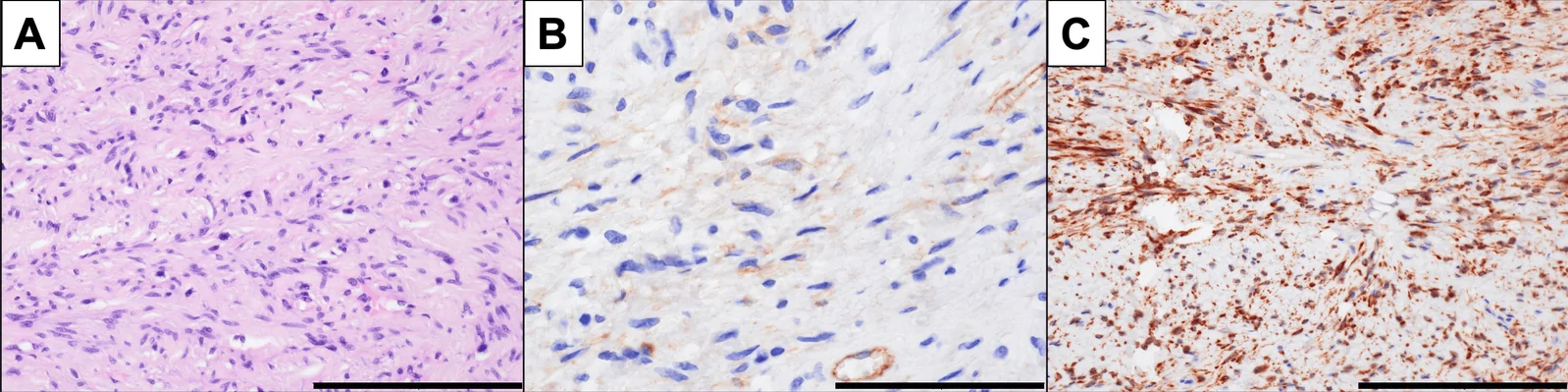
Histopathological and immunohistochemical examinations (A) Hematoxylin and eosin staining showing spindle-shaped cells arranged in a patternless pattern with moderate cellularity and prominent vascularity. (B) Immunohistochemical staining demonstrating diffuse positivity for CD34. (C) Immunohistochemical staining demonstrating diffuse positivity for Bcl-2. Scale bar = 200 μm.

Histopathological examination revealed spindle-shaped cells arranged in a “patternless pattern” architecture with moderate cellularity and prominent vascularity (Figure 3, Panel A). Immunohistochemical staining revealed that the tumor cells were positive for CD34 (Figure 3, Panel B) and Bcl-2 (Figure 3, Panel C), and negative for S-100, desmin, alpha-smooth muscle actin (α-SMA), c-kit, and CD99. Although STAT6 immunostaining was not performed, the morphological and immunophenotypic features were consistent with SFT. Based on these findings, a final diagnosis of mesorectal SFT was established. No recurrence was observed at the six-month follow-up period. Given the potential for late recurrence, the patient is currently undergoing surveillance with contrast-enhanced CT every six months during the first two postoperative years, followed by annual imaging thereafter.

## Discussion

SFT is a rare soft tissue tumor with an estimated incidence of 2.8 cases per 100,000 individuals [4]. While SFTs can occur in various anatomical sites, the pleura remains the most common location. A recent study reported that 34% of SFTs originate in the abdominopelvic region [5]. Nevertheless, mesorectal SFT is extremely rare, and its clinical features and optimal management strategies have not been well established. Our literature review identified only eight previously reported cases of mesorectal SFT (Table 1). Including the present patient, a total of nine cases have been reported, comprising five males and four females, with a median age of 52 years (range, 27-69 years). All cases were treated by surgical resection; three cases required rectal resection, while six underwent local tumor excision. During a median follow-up period of 24 months, only one case of recurrence was reported [6].

**Table 1:** Reported cases of solitary fibrous tumor from the mesorectum F: Female; M: Male; N/A: Not applicable; APR: Abdominoperineal resection; LAR: Low anterior resection; HAR: High anterior resection; TAMIS: Transanal minimally invasive surgery.

Case	Authors	Age	Sex	Symptoms	Size (cm)	Surgical procedure	Surgical approach	Follow-up (months)	Recurrence
1	Soda et al. [10].	27	F	Constipation, urinary retention	16	APR	Open and transsacral	24	No
2	Teshima et al. [9].	36	M	Constipation, urinary retention	15.5	Tumor resection	Open	26	No
3	Troja et al. [14].	60	M	Weight loss	9	Tumor resection	Transsphincteric	N/A	N/A
4	Kawamura et al. [12].	56	F	No	12	Tumor resection	Laparoscopy	18	No
5	Yuza et al. [7].	46	F	Hypoglycemia	17	LAR	Open	24	No
6	Chiu et al. [6].	69	M	N/A	N/A	Tumor resection	N/A	48	Peritoneal sarcomatosis
7	Moriuchi et al. [11].	49	F	No	8	HAR	Open	8	No
8	Ströse et al. [13].	52	M	Abdominal pain	2.3	Tumor resection	TAMIS	40	No
9	Present case	62	M	No	3.5	Tumor resection	Laparoscopy	6	No

Patients with SFT are typically asymptomatic until the tumor reaches a large size sufficient to compress the adjacent organs. Rare paraneoplastic syndromes have been associated with SFT, including Doege-Potter syndrome (hypoglycemia caused by a tumor overproducing insulin-like growth factor 2) and Pierre-Marie-Bamberger syndrome (hypertrophic osteoarthropathy) [7,8]. In previously reported cases of mesorectal SFT, patients presented with abdominal pain, constipation, or urinary retention, while three of the nine reported patients (33%) were asymptomatic. In this case, the tumor was small, which explains the lack of clinical symptoms.

In pelvic mesenchymal tumors, biopsy may be technically difficult and associated with risks such as bleeding, infection, or tumor seeding. Fine-needle aspiration biopsies often yield insufficient tissue for a definitive diagnosis, primarily due to the heterogeneous distribution of cellular density within the tumor [9]. In our case, preoperative imaging demonstrated a well-circumscribed tumor without invasion into adjacent organs. We avoided biopsy because of the deep pelvic location and potential risk of tumor dissemination or bleeding.

Complete surgical resection remains the standard treatment for SFT. Previous cases of large mesorectal SFT often described the necessity of laparotomy due to technical difficulty and the risk of massive hemorrhage [6,7,10,11]. In contrast, several reports have demonstrated the feasibility of laparoscopic surgery, transanal minimally invasive surgery (TAMIS), and transsphincteric resection [12-14]. In our case, the relatively small tumor size and the absence of local invasion allowed a safe laparoscopic resection without the need for rectal excision or significant blood loss.

A definitive diagnosis of SFT is confirmed by histopathology and immunohistochemistry. SFT is characterized histologically by spindle-to-oval cells arranged in a "patternless pattern" [5]. Immunohistochemically, STAT6 is considered a highly sensitive and specific marker; however, CD34 and Bcl-2 positivity also support the diagnosis [1]. The absence of STAT6 immunohistochemistry represents a limitation of this case. Although the diagnosis was supported by characteristic histological features together with the immunophenotype, the diagnosis of SFT would have been strengthened by STAT6 positivity.

The WHO Classification of Tumors of Soft Tissue and Bone categorizes SFT as a mesenchymal neoplasm with intermediate (rarely metastasizing) malignant potential [15]. Local or distant recurrence occurs in 10%-30% of cases [3]. Currently, there is no established standard treatment for recurrent or metastatic disease, and SFT is generally considered chemoresistant [16]. One reported case of mesorectal SFT with peritoneal metastasis after tumor resection was treated by peritoneal lavage with 10 L of saline in the abdominopelvic cavity and cytoreductive surgery, achieving a disease-free survival of 28 months [6]. The Demicco risk assessment model incorporates patient age, tumor size, mitotic activity, and tumor necrosis to estimate the risk of metastasis in SFT [17]. In the present case, the small tumor size (35 mm), absence of necrosis, and lack of increased mitotic activity suggest a relatively low risk of recurrence, although long-term surveillance remains warranted because late recurrence has been reported even more than a decade after initial resection [18].

## Conclusions

We report a rare case of mesorectal SFT successfully treated by laparoscopic tumor resection. Although limited to a single case, our experience suggests that laparoscopic resection may be a feasible treatment option in carefully selected patients. Long-term postoperative surveillance remains essential because of the potential for late recurrence.
